# Transient Magnetothermal Neuronal Silencing Using the Chloride Channel Anoctamin 1 (TMEM16A)

**DOI:** 10.3389/fnins.2018.00560

**Published:** 2018-08-14

**Authors:** Rahul Munshi, Shahnaz M. Qadri, Arnd Pralle

**Affiliations:** Department of Physics, University at Buffalo, Buffalo, NY, United States

**Keywords:** magnetothermal, magnetogenetic, remote silencing, anoctamin 1, chloride channel

## Abstract

Determining the role and necessity of specific neurons in a network calls for precisely timed, reversible removal of these neurons from the circuit via remotely triggered transient silencing. Previously, we have shown that alternating magnetic field mediated heating of magnetic nanoparticles, bound to neurons, expressing temperature-sensitive cation channels TRPV1 remotely activates these neurons, evoking behavioral responses in mice. Here, we demonstrate how to apply magnetic nanoparticle heating to silence target neurons. Rat hippocampal neuronal cultures were transfected to express the temperature gated chloride channel, anoctamin 1 (TMEM16A). Spontaneous firing was suppressed within seconds of alternating magnetic field application to anoctamin 1 (TMEM16A) channel expressing, magnetic nanoparticle decorated neurons. Five seconds of magnetic field application leads to 12 s of silencing, with a latency of 2 s and an average suppression ratio of more than 80%. Immediately following the silencing period spontaneous activity resumed. The method provides a promising avenue for tether free, remote, transient neuronal silencing *in vivo* for both scientific and therapeutic applications.

## Introduction

Spatially and temporally regulated signaling in complex brain circuits controls behavior, emotions and other brain functions. Tools to modulate specific network components and connections are absolutely crucial in the study of the functional connectivity of these circuits. In the past decade, electrical, optical, chemical, and magnetic tools to activate specific neurons, deep in the brain have been developed (Warden et al., 2014; Deisseroth, 2015; Kim et al., 2017). However, understanding the role of a specific neuron in a network requires selective, temporally controlled reversible activation and silencing of that component (Nomura et al., 2015). In our earlier work we had presented magnetothermal activation as a remote tether-free neuronal activation tool (Munshi et al., 2017). Here we introduce magnetothermal neuronal silencing as a robust temporally controlled silencing tool.

In the recent times, a variety of neuronal silencing strategies have been adapted (Slimko et al., 2002; Chow et al., 2010; Okazaki et al., 2012; Wiegert et al., 2017). Silencing is typically achieved by holding the membrane potential sufficiently negative to suppress action potential firing (Hodge, 2009). Pharmacological and chemogenetic approaches, like those using engineered small molecules to manipulate G-protein signaling pathways (Armbruster et al., 2007; Stachniak et al., 2014) or ligand gated ion channels to subdue action potentials (Slimko et al., 2002; Lerchner et al., 2007; Vardy et al., 2015; Weir et al., 2017) have slow on and off kinetics (minutes – hours).

Optogenetic tools employ modified opsins as light driven inward chloride pumps or outward proton pumps (Chow et al., 2010; Raimondo et al., 2012; Sudo et al., 2013; Okazaki et al., 2014). More recently, a chloride conducting channelrhodopsin was developed (Wietek et al., 2014, 2015; Govorunova et al., 2015, 2017), which uses a light gated ion channel based silencing mechanism. Photothermally induced hyperpolarization in wild type neurons has been achieved by optically heating nanomaterial absorbers on the cell surface, such as polymers (Feyen et al., 2016) or plasmonic gold nanorods (Yoo et al., 2014). As the photothermal approaches rely on an endogenous response of the neurons, they do not provide any cell specificity. While optogenetic and photothermal approaches provide a fast response (milliseconds to seconds), they require an invasive light delivery mechanism for deep brain modulation. To overcome the tissue penetration issue, upconversion nanoparticles, which absorb in the near infrared region and emit in the wavelength range of inhibitory opsins (Lin et al., 2017; Chen et al., 2018) are being explored to remotely silence deep brain neurons (Lin et al., 2018).

**FIGURE 1:**
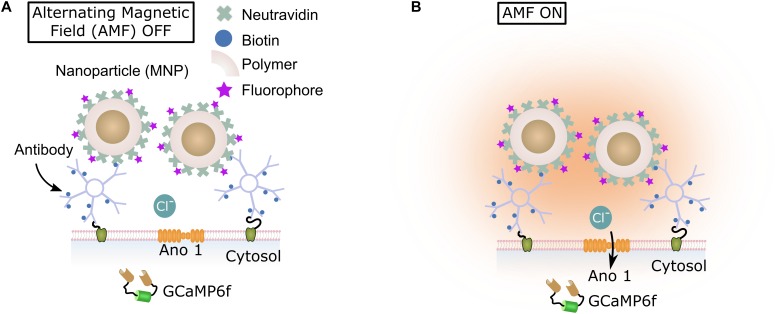
Magneto-thermal silencing scheme. **(A,B)** Illustrate the principle of magnetogenetic silencing. Magnetic nanoparticles (brown) were encapsulated in polymer (PMMA). Fluorescent dye (Alexa Fluor 647) molecules were bound to Neutravidin molecules (green; 5:1 molar ratio). The Neutravidin molecules were covalently attached to the nanoparticle polymer layer. These particles were then attached to the neuronal cell membranes via biotin-avidin bonding formed with the membrane attached biotinylated IgM antibodies. When exposed to alternating magnetic fields (AMF), the particles heat, opening the temperature gated membrane Ano1 channels **(B)**. This causes an influx of Chloride ions, leading to membrane hyperpolarization.

Here, we introduce magnetothermal silencing, using the heat generated by superparamagnetic nanoparticles to activate the thermosensitive chloride channel anoctamin 1 (TMEM16A) (Yang et al., 2008; Cho et al., 2012; Paulino et al., 2017a,b). We co-transfected membrane protein Ano1/TMEM16A and cytosolic calcium indicator protein GCaMP6f (Chen T.W. et al., 2013) in rat hippocampal cultures. Polymer encapsulated superparamagnetic nanoparticles were bound to the surface of neurons via A2B5 antibodies. When exposed to alternating magnetic fields, these particles heated up, raising the membrane temperature (Huang et al., 2010; Munshi et al., 2017). The elevated membrane temperature causes Ano1/TMEM16A channels to open, leading to an inhibition of calcium influx, recorded by the GCaMP6f signal (**Figure 1**). Within seconds of removing the magnetic field, membrane temperature returned to physiological value, and spontaneous calcium activity resumed. As Ano1/TMEM16A is a mammalian chloride channel (Huang et al., 2009; Cho et al., 2012; Terashima et al., 2013), no further protein engineering is necessary to express it in mammalian neurons (Hodge, 2009; Berndt et al., 2014; Wietek et al., 2015). The alternating magnetic fields used in magnetothermal silencing penetrate tissues easily (Oleson, 1982; Chen et al., 2015; Munshi et al., 2017). Only cells expressing Ano1/TMEM16A channels and having membrane bound nanoparticles are silenced, while the adjoining cells remain unaffected. Thus, the method allows to remotely and temporarily disengage a neuronal subpopulation from a brain circuit to investigate their importance for that circuit.

## Materials and Methods

### Rat Hippocampal Neuronal Cultures

Primary rat hippocampal neurons were harvested from E18 rat fetuses, from Timed Pregnancy female Sprague Dawly rats (Harlan), following a modified standard protocol (Brewer, 1997). All animal protocols were approved by the Animal Care and Use Committee at University at Buffalo, State University of New York. Neurons were cultured on 12 mm poly-L-lysine (Sigma-Aldrich, P6282) coated coverslips at 70–75% confluence, placed in 24-well cell culture plates, housed in a sterile incubator at 37°C with 5% CO_2_. Neurobasal medium (Thermo Fisher Cat. # 12348017) was used with added 2% v/v Glutamax (Thermo Fisher Cat. # 35050061), 2% v/v B-27 supplement (Thermo Fisher Cat. # 17504044) and 1% v/v Penicillin-Streptomycin (Thermo Fisher Cat. # 15140122). Every 3 days, half of the medium was replaced with fresh medium.

### Transfection of Hippocampal Neurons

Plasmids encoding Anoctamin 1 with an mCherry marker (Ano1/TMEM16A-mCherry) and GCaMP6f were introduced into the hippocampal neurons using Calcium Phosphate method, following published protocols (Jiang and Chen, 2006). A day prior to the procedure, coverslips were transferred from 24 well plates to 35 mm dishes (4 in each dish, 70–75% confluent), filled with 1.5 ml sterile filtered, conditioned medium (medium that had been on the cells for 2–3 days). For each dish (1.5 ml medium) a total of 4–7 μg DNA was used (depending on prior titration results). For co-transfection, 4 μg Ano1/TMEM16A-mCherry p-DNA was mixed with 3 μg GCaMP6f p-DNA in DI water and 9 μl of CaCl_2_ solution (2M) was dropwise added. Prior to the addition of CaCl_2_, the volume of water was adjusted, so that the final volume of the mixture was 45 μl. After thoroughly mixing, the mixture was dropped into 45 μl of 2x HEPES buffer (NaCl, 274 mM; KCl, 10 mM; Na_2_HPO_4_.7H_2_O, 1.4 mM; D-glucose, 15 mM; HEPES (free acid) 42 mM. pH = 7.06). Following a 15 min precipitation period in the dark at room temperature, the mixture was added to the dish containing neurons (in 1.5 mL medium) with evenly distributed drops. Following a 15–20 min incubation (in the 37°C, 5% CO_2_ incubator) the dishes were aspirated with quenching medium, MEM (Thermo Fisher Cat. # 11575032) with 10% v/v FBS (Thermo Fisher Cat. # 16000044), was finally placed in conditioned medium (1.5 ml per 35 mm dish housing four 12 mm cover slips). All transfections were performed on DIV 4–7, with the highest yield being generally from DIV 5.

### Preparation of Neurons for Silencing and Imaging

After transfection, the neuronal cultures were placed in incubator (37°C, 5% CO_2_) for next 48–72 h before experiment. 24 h prior to the experiment, 1 μM TTX (Sigma-Aldrich, 4368-28-9) was added to the culture medium. For imaging, the neurons were placed in imaging bath solution [NaCl 145.0, CaCl_2_ 2.0, MgCl_2_ 1.0, KCl 2.5 (or 4.0 for high K^+^), HEPES 10.0, Glucose 20.0 (all in mM) at pH 7.34 and an osmolality of 310–315 mOsmole/L].

Synthesized superparamagnetic core-shell Co-Mn-Ferrite nanoparticles (MNP) coated with PMA (poly-isobutylene-maleic anhydride) were functionalized by covalently attaching Neutravidin (Thermo Fisher Cat. # 31000) to the PMA layer (Lin et al., 2008; Zhang et al., 2015). Prior to this step, dye molecules (Thermo Fisher Cat. # A37573) were attached to the neutravidin molecules (Munshi et al., 2017; Castellanos-Rubio et al., 2018).

Cells growing on 12 mm cover slips were transferred to a Delrin imaging chamber (ALA MS-512DWPW) and 200 μL of the imaging bath solution was immediately added above the cells. Ten-minute incubation (at 37°C) with 1.5 μg of biotinylated A2B5 antibody (Invitrogen Cat. # 433110) was done (antibody dilution 1:200 – 1:100, for fast staining). The bath solution was then aspirated and replaced with buffer solution containing 2 μg functionalized MNPs in 200 μl of fresh bath solution and incubated for 10 min at 37°C. This incubation period resulted in optimal attachment of the nanoparticles to the antibodies, though biotin-avidin bonding. After this period, the unbound nanoparticles were washed out, leaving just a layer of nanoparticles decorated over the entire cytosolic membrane. The schematic diagram in **Figure 1** illustrates the nanoparticle attachment mechanism in detail.

### Live Cell Imaging Under Alternating Magnetic Fields

Alternating magnetic fields (AMF) were generated by a 5 mm diameter, 5 turn water cooled copper coil driven by a 7.5 kW AC power supply (MSI Automation). The coil surrounded the imaging dish (ALA Scientific instruments, MS-512DWPW) placed over the microscope (Zeiss Axio Observer A1 M) objective (Zeiss, working distance, 0.71 mm). AMF heats the metallic objective by producing eddy currents, causing focus drift and optical aberrations. To correct these in the real time, we used a fast, customized piezoelectric autofocus system (Motion X Corporation, 780 nm laser interferometer based). A custom-made microenvironment chamber was used to enclose the entire microscope with a stable ambient temperature. Precise control over the bath temperature was achieved by an inline solution heater (Warner instruments, controller TC324B), connected to a homemade perfusion system. HBO 200 lamp with appropriate filters were used for illuminating the fluorescent dyes [GCaMP6f: ex, FF02-472/30; DIC, FF495-Di03; em, FF01-525/30. Alexa Fluor 647: ex, FF01-635/18; DIC, Di02-R635; em, FF01-680/42 (Filters from Semrock)]. Fluorescence data were recorded at 10 Hz, using an Andor NEO sCMOS camera controlled by microManager software (Edelstein et al., 2010).

### Image Processing and Intensity Normalization

Data were acquired as 16-bit gray scale image stacks. The average fluorescence intensities of an ROI (regions of interest) in the fluorescence microscopy images were extracted using FIJI (Fiji Is Just ImageJ). For this, intensity-based thresholding was performed by converting the non-contributing pixels (with background intensity values) to NaN (not a number). The intensity versus time data were then further processed using IgorPro (WaveMetrics).

The ROI intensity data contained fluorescence signal, offset by dark background noise. The dark noise is the average signal recorded by the camera under no illumination conditions. All experiments were done under similar ambient light conditions and the dark noise value, generally a function of the camera exposure time and binning deviated little from experiment to experiment. The constant dark noise value was subtracted from all data. Bleach correction was only performed, if the baseline showed substantial bleaching. In those cases, the baseline was fitted with an exponential function and a modified signal was obtained according to Equation (1).

$$F(t)=F^{\prime }(t)+\left(F^{\prime }\left(0\right)-{F^{\prime }}_{fit}\left(t\right)\right)$$

Here, *F′*(*t*) is the signal values obtained after dark noise subtraction and $F_{fit}^{\prime }$(*t*) is the corresponding exponential fit function. Data thus modified contained calcium peaks over a constant baseline. The data was then normalized and converted to percentage change in fluorescence. The percentage change in fluorescence intensity was given by Equation (2).

$$\frac{\Delta F\left(t\right)}{F_{0}}=\frac{F(t)-F_{0}}{F_{0}}\times 100$$

Where _F_0__ is the bleach corrected baseline ROI intensity and *F*(*t*), the intensity at any time *t*.

### Reconstruction of AP Firing Pattern From GCaMP6f Calcium Signals

#### Single Spike Estimation

**FIGURE 2:**
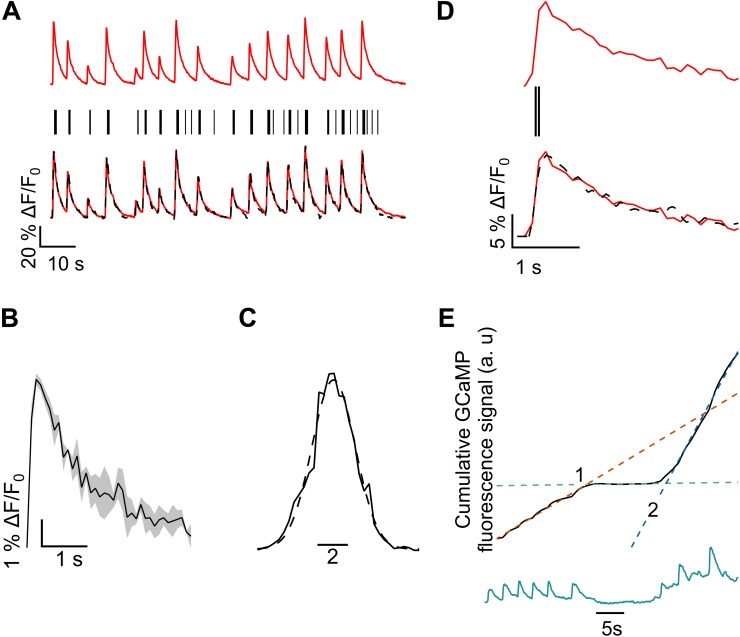
Methodology for spike estimation and latency calculation. **(A)** (Top) Bleach corrected, normalized GCaMP6f data. (Middle) Predicted action potential events from GCaMP6f trace (black sticks) and (bottom) the overlay of regenerated trace obtained from convolving predicted action potential events with single AP calcium peak (black, broken) with the normalized GCaMP6f trace. **(B)** The average (*n* = 3 peaks) signal from three smallest peaks recorded from the same neuron in a single recording. **(C)** Histogram of the residual of the GCaMP6f trace and the regenerated trace in **(A)**, fitted with a Gaussian curve. The sigma of the fit was 1.42 ± 0.05. **(D)** Extension of **(A)**, showing a horizontally magnified view of GCaMP6f peak fitting. Convolution of the two estimated APs (black bars) with the average single peak profile **(B)** gives the reconstructed GCaMP6f peak (black broken). **(E)** Representative numerical integration (top, black) of the GCaMP6f plot (bottom, green). Suppression in firing is indicated by a reduction in slope of the integration plot. Dotted lines show linear fits of three distinct sections of the trace [red: before suppression (left of 1), green: during suppression (between 1 and 2), blue: after resumption, following suppression (right of 2)]. Slopes of these lines give average rate of Ca^2+^ influx, during the indicated periods. The points of intersection of these lines give the times corresponding to the beginning (1) and ending (2) of suppression.

The sensitivity of GCaMP6f enables the detection of single action potentials. However, as the time between APs in a spike train is often shorter than the slow decay of GCaMP6f, it is challenging to isolate individual APs (Chen T.W. et al., 2013). To extract AP events from a GCaMP6f fluorescence signal (Ca^2+^ peaks) (**Figure 2A**, red), we first identified all single peaks from the baseline corrected and intensity normalized data.

A GCaMP6f peak was considered to have resulted from a single AP spike if the peak was distinctly one of the smallest in the bleach corrected, normalized data and if the peak.

**FIGURE 3:**
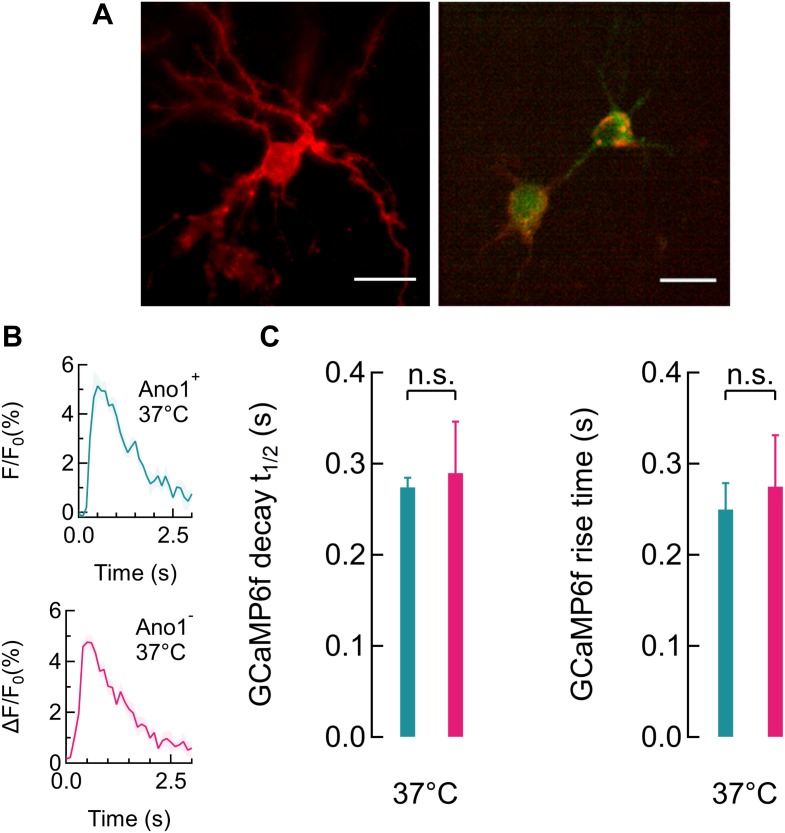
Ano1/TMEM16A expression does not alter GCaMP6f peaks. **(A)** Ano1/TMEM16A expression in rat hippocampal neurons, visualized with the mCherry tag (red) and GCaMP6f fluorescence (green) overlay (right). **(B)** Average single gCAMP6f peaks recorded in Ano1/TMEM16A^+/-^ neurons (top and bottom, respectively) at 37°C, respectively (*n* = 4). **(C)** GCaMP6f peak rise times for Ano1/TMEM16A^+/-^ neurons were found to be 0.25 ± 0.03 s and 0.28 ± 0.05 s, respectively. Peak decay half-life times for Ano1/TMEM16A^+/-^ neurons were 0.27 ± 0.01 s and 0.29 ± 0.06 s, respectively. No significant change in peak characteristics was found (*n* = 4, all cases). Color scheme for Ano1/TMEM16AC^+/-^ data follows the scheme used in **(B)**.

The assignment of the smallest GCaMP6f peaks as being by a single AP is an assumption well supported by the amplitude, rise time and decay times of the corresponding profiles (**Figures 2B**, **3B**) which agree well with published single AP GCaMP6f recordings (Chen T.W. et al., 2013; Park et al., 2013; Deneux et al., 2016). For a small number of near simultaneous APs (like those occurring during bursts), the signal amplitude of GCaMP6f is approximately linearly proportional to the number of APs (Chen T.W. et al., 2013; Park et al., 2013; Deneux et al., 2016). The signal decay time is an intrinsic property of the Ca^2+^ sensor resulting from the unbinding of Ca^2+^ and does not reflect the actual shape of the Ca^2+^ spike in the cell. Also, subsequent reconstruction of the GCaMP6f signal using convolution of this single spike profile estimate showed excellent fitting over large datasets. Even if the observed single signal actually was caused by multiple APs, the overestimation would not affect the relative change in firing rate during silencing (Yaksi and Friedrich, 2006; Pnevmatikakis et al., 2016).

#### AP Event Localization

To generate the AP spike train, or time course of AP events, a binary trace of duration equal to intensity rose about 5% above the baseline. All such isolated spikes were pooled to an average GCaMP6f peak, corresponding to a single AP firing. The average GCaMP6f peak profile data was then interpolated linearly to reduce the time interval between data points from 0.10 s (image acquisition exposure time) to 0.01 s (**Figure 2B**).

An estimated spike train was generated as binary trace of duration equal to the original data but sampled at 100 Hz frequency (10 Hz for original data) with *ones* at the estimated location of the AP spikes (**Figure 2A**, bottom trace). To quantify the precision of this spike train estimate, the average single-AP GCaMP6f profile (from the previous section, **Figure 2B**) was convolved with the binary trace and compared to the original data trace (**Figures 2A, D**). Convolution inserts a single-AP GCaMP6f profile at the location of each estimated AP spike. An overlay of the bleach corrected and normalized GCaMP6f data (red) and its corresponding convolution-based reconstruction (black broken) is shown in **Figure 2A**. A histogram of the residuals of the original and the reconstructed traces was fitted to a Gaussian function (**Figure 2C**). The estimated locations of the spikes were adjusted until the overall position error (σ) was less than 2% (Vogelstein et al., 2010; Park et al., 2013; Pnevmatikakis et al., 2016). The temporal error in AP estimation is determined by GCaMP6f kinetics as well as error in fitting.

#### Average AP Firing Rate Estimation

Average firing rates were calculated either directly from the reconstructed spike train, or estimated from Ca^2+^ peaks found automatically, using IgorPro’s Multipeak Fitting 2.0 package. After baseline removal, each Ca^2+^ peak was automatically fitted and the peak height and location were recorded. The peak heights were then normalized to the lowest peak height value, and rounded to the closest integers. This technique gives estimates of number of AP spikes constituting each Ca^2+^ peak, binned over a given time period, with error within GCaMP6f’s resolution limit.

### Spiking Independent Quantification of Latency, Extent and Duration of Silencing

It is possible to quantify latency, extent and duration of silencing without reconstructing the AP spiking train. To calculate a sliding average of the calcium activity, we numerically integrated (trapezoidal) the normalized, bleach corrected and baseline subtracted GCaMP6f data. The integration curve serves as an indicator of cumulative intracellular Ca^2+^ influx. A reduction of the slope of the linear fit of the integrated GCaMP6f signal curve indicates a decline of Ca^2+^ activities, while an increased slope indicates increasing activity (**Figure 2E**). A section of the integration curve with reduced average slope indicated a period of suppressed activities, compared to other sections with steeper slopes. In **Figure 2E** integration (black) of the GCaMP6f signal (green, bottom) clearly captures the three distinct phases of Ca^2+^ activity. The first section shows GCaMP6f signal corresponding to spontaneous firing, the second section shows a period of inactivation, while the third section shows a resumption in firing. Linear fits (broken, overlaid) corresponding to each section of the integration plot intersect at points of transition of Ca^2+^ influx rates (points 1 and 2, **Figure 2E**). The point of intersection of the fitting lines of the first and the second sections determined the starting time (point 1), while the intersection of the fitting lines of the second and third sections gave the ending time of the silencing period (point 2). Latency was given as the temporal difference between the start of the AMF and the starting time of silencing (point 1). The duration of silencing was given by the difference between the ending and the starting times of silencing (point 2 – point 1). Latency and duration were calculated from the times obtained from individual plots, and later averaged.

All numerical data were processed in IgorPro 7.0.

## Results

### Thermal Suppression of Ca^2+^ Influx in Ano1/TMEM16A^+^ Neurons

We first determined if spontaneously firing Ano1/TMEM16A^+^ neurons could be significantly inactivated at higher temperatures. For this, we co-transfected rat hippocampal neurons with plasmid DNAs for mAno1/TMEM16A-mCherry and the genetic calcium indicator, GCaMP6f. Experiments were performed 48–72 h after transfection. To demonstrate successful silencing it was necessary to begin the experiment with an actively firing culture. The cultured neurons were treated with 1 μM TTX, 24 h prior to experiments. Right before the experiments, the TTX was washed out by perfusion (2 ml/min) with high K^+^ imaging buffer. Experiments were started only after spontaneous Ca^2+^ activity was observed, following the TTX wash. Chronic TTX incubation blocks spontaneous activity in the culture and washing increases the excitability of the pyramidal neurons (Desai et al., 1999; Hochbaum et al., 2014). Cells expressing Ano1/TMEM16A were identified by the mCherry marker (**Figure 3A**). GCaMP6f fluorescence signal captures cytosolic Calcium transients, resulting from membrane depolarization (Chen T.W. et al., 2013). The average GCaMP6f peak, corresponding to a single action potential (AP) firing was indistinguishable between neurons with and without Ano1/TMEM16A (**Figure 3B**). TTX incubation followed by high K^+^ buffer washing produced fast firing and regular GCaMP6f peaks were recorded for up to 1 h. No difference in firing duration was seen between Ano1/TMEM16A^+/-^ cells at 37°C. At 37°C, the rise times (duration until calcium peak is reached from the baseline) were 0.275 ± 0.048 s and 0.250 ± 0.029 s (*p* = 0.674), and the half-decay time for the Ca^2+^ decay were 0.274 ± 0.011 s and 0.290 ± 0.055 s (*p* = 0.801) in Ano1/TMEM16A^+/-^ neurons, respectively (**Figure 3C**).

**FIGURE 4:**
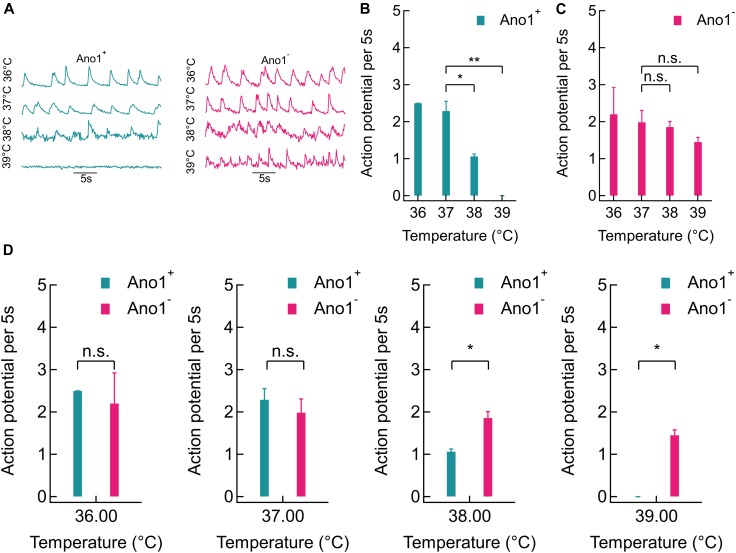
Thermal silencing of Ano1/TMEM16A^+^ hippocampal neurons. **(A)** Comparison of representative GCaMP6f traces between Ano1/TMEM16A^+/-^ neurons at 36, 37, 38, and 39°C (left and right, respectively). **(B,C)** Action potential firing rate (per 5 s) comparison in Ano1/TMEM16A^+/-^ neurons at various bath temperatures. Significant suppression in firing rate was observed at 38°C (*p* = 0.0163) and 39°C (*p* = 0.0032) in Ano1/TMEM16A^+^ neurons, compared to 37°C (*T*-test). In Ano1/TMEM16A^-^ neurons similar comparison yielded non-significant changes (*p* = 0.7067 and *p* = 0.1682 at 38 and 39°C, respectively). **(D)** Firing rates of Ano1/TMEM16A^+^ and Ano1/TMEM16A^-^ neurons were not significantly different at 36°C (*p* = 0.6628) or 37 °C (*p* = 0.4526), while firing rates at 38 and 39°C varied significantly between Ano1^+^ and Ano1^-^ neurons (*p* = 0.0227 and 0.0051, respectively). ^∗^*p* < 0.05, ^∗∗^*p* < 0.005.

Representative GCaMP6f fluorescence intensity traces recorded from Ano1/TMEM16A^+/-^ cells at various steady bath temperatures (36, 37, 38^,^ and 39°C) are shown in **Figure 4A**. A sharp reduction in GCaMP6f peaks was seen in Ano1/TMEM16A^+^ traces at 38°C and more significantly at 39°C, where the peaks completely disappeared (**Figure 4B**). On the other hand, in Ano1/TMEM16A^-^ cells, no such striking suppression in Ca^2+^ activities was observed (**Figure 4C**). To quantify thermal suppression, we calculated AP firing rates from GCaMP6f fluorescence intensity traces by deconvolving the calcium transients. The 5 s AP firing rates in Ano1/TMEM16A^+^ cells decreased significantly from 37 to 39°C: from 2.29 ± 0.26 at 37°C to 1.06 ± 0.06 at 38°C and 0 at 39°C [*p* = 0.016 at 38°C (*n* = 4) and *p* = 0.003 at 39°C (*n* = 5) compared to 37°C, unpaired *T*-test] (**Figure 4B**). On the other hand, the AP firing rate of Ano1/TMEM16A^-^ neurons did not change significantly over the same temperature range [AP rates averaged over 5 s (*n* = 5) were 2.20 ± 0.72 at 36°C, 1.98 ± 0.32 at 37°C, 1.85 ± 0.15 at 38°C and 1.45 ± 0.13 at 39°C]. The *p*-values of rates, compared to 37°C was 0.707 at 38°C and 0.168 at 39°C, obtained by unpaired *T*-test (**Figure 4C**).

The firing rates of Ano1/TMEM16A^+^ neurons were indistinguishable from that of Ano1/TMEM16A^-^ neurons at 36 and 37°C (*p* = 0.663 and 0.453, respectively). However, at 38 and 39°C the spiking rates deviated significantly (*p* = 0.0227 and 0.0051, respectively) (**Figure 4D**). Furthermore, Ano1/TMEM16A^-^ cells fired even at 40°C at 1.38 ± 0.61 APs per 5 s. The extracellular calcium concentration was fixed at 2 mM for all the experiments. This establishes that in Ano1/TMEM16A overexpressing neurons, 1–2°C elevation above the physiological temperature is sufficient to disrupt homoeostasis and cause significant suppression in action potential firing, unlike its wild type counterpart.

### Heating Neuronal Plasma Membrane With Nanoparticles

**FIGURE 5:**
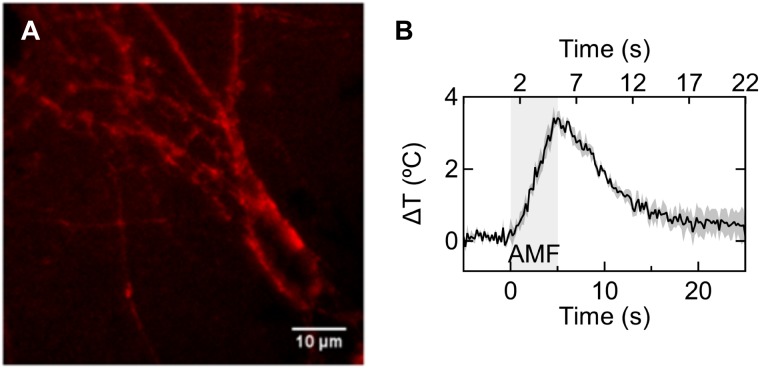
Nanoparticle heating on the neuronal membrane. **(A)** Fluorescence micrograph showing magnetic nanoparticles coated with Alexa fluor-647 labeled NeutrAvidin attached to the neuronal membrane in a rat hippocampal culture via biotinylated anti-A2B5 antibody. **(B)** Plot shows average temperature rise in magnetic nanoparticle decorated membrane under 5 s magnetic field (*n* = 3). Bottom axis shows time relative to AMF start, and top axis shows 5 s time bins when significant temperature points are achieved (e.g., 2–7 s = 1–3°C, 7–12 s = 3–1°C, 17–22 s <1°C above the starting temperature).

For remote magnetothermal silencing of Ano1/TMEM16A^+^ neurons, we targeted superparamagnetic nanoparticles to the cell membrane as local heating agents. Core-shell nanoparticles (MNP) with a 6.7 ± 1.0 nm MnFe_2_O_4_ core and a CoFe_2_O_4_ shell, with a total inorganic diameter of 12.9 ± 1.4 nm were encapsulated in PMA [dodecyl-*grafted*-poly-(isobutylene-*alt*-maleic-anhydride)] (Lin et al., 2008; Zhang et al., 2015). The specific loss in power (SLP) of these particles, suspended in water, was 553 ± 10 W/g in a 37 kA/m alternating magnetic field (AMF), driven at 412.5 kHz. The outer PMA layer was functionalized with Neutravidin, pre-modified with Alexa Fluor 647 dye. To specifically target nanoparticles to the neuronal cell membrane, we briefly incubated neurons first with biotinylated A2B5 antibodies. Then the Neutravidin-dye tagged nanoparticles were added. After washing, only the membrane bound nanoparticles remained (**Figures 1**, **5A**), effectively creating a shape conforming array of nanoscopic heat sources on the cell membrane (Huang et al., 2010; Munshi et al., 2017). Under alternating magnetic fields, these nanoparticles heated the membrane enough to open Ano1/TMEM16A channels.

To measure the temperature change of the magnetothermally heated cell membrane, we employed autofocus stabilized epifluorescence microscopy under alternating magnetic fields. Time series imaging of the fluorescent tag (Alexa Fluor 647) of the membrane bound nanoparticles enabled measuring temperature changes close to the membrane. In the temperature range of interest, the fluorescence emission intensity of the Alexa Fluor 647 fluorophore decreases approximately linearly with increasing temperature. The rate of normalized intensity change versus temperature was -0.46% °C^-1^. Five seconds of AMF application (28.87 ± 1.03 kA/m 412.5 kHz) was sufficient to raise the temperature by 3.42 ± 0.17°C (**Figure 5B**), corresponding to a heating rate of 0.75 ± 0.03°C/s. Cooling of the membrane starts instantaneously with the turning off of the AMF. With an offset of 2 s from the start of the AMF, we binned time into 5 s intervals. The approximate absolute temperature limits in the time windows were 38–40°C during 2–7 s, 38–40°C during 7–12 s, and 37–38°C during 17–22 s. These time points are marked on the top axis of **Figure 5B**, for visual guidance. The starting temperature in all experiments was 37°C.

### Magnetothermal Silencing of Ano1/TMEM16A^+^ Neurons

To demonstrate transient magnetothermal, Ano1/TMEM16A mediated silencing of neuronal activities, we co-transfected cultured, mature rat hippocampal neurons with mAno1/TMEM16A-mcherry and GCaMP6f plasmid DNAs, 48 h prior to the experiment. 1 μM TTX was added to the culture medium 24 h prior to the experiment. Before the experiment, Alexa Fluor 647 labeled magnetic nanoparticles (MNPs) were bound to the neuronal membrane, using biotinylated A2B5 antibodies. Ano1/TMEM16A^+^ neurons were identified by mCherry marker. Cells were washed with high K^+^ imaging buffer to remove any remaining TTX molecules as well as any unbound antibodies or MNPs. Perfusion with the high K^+^ imaging buffer was started prior to imaging and was maintained at 2 ml/minute, while the temperature of the sample holder was held at 37°C by a solution inline heater driven by a temperature controller.

**FIGURE 6:**
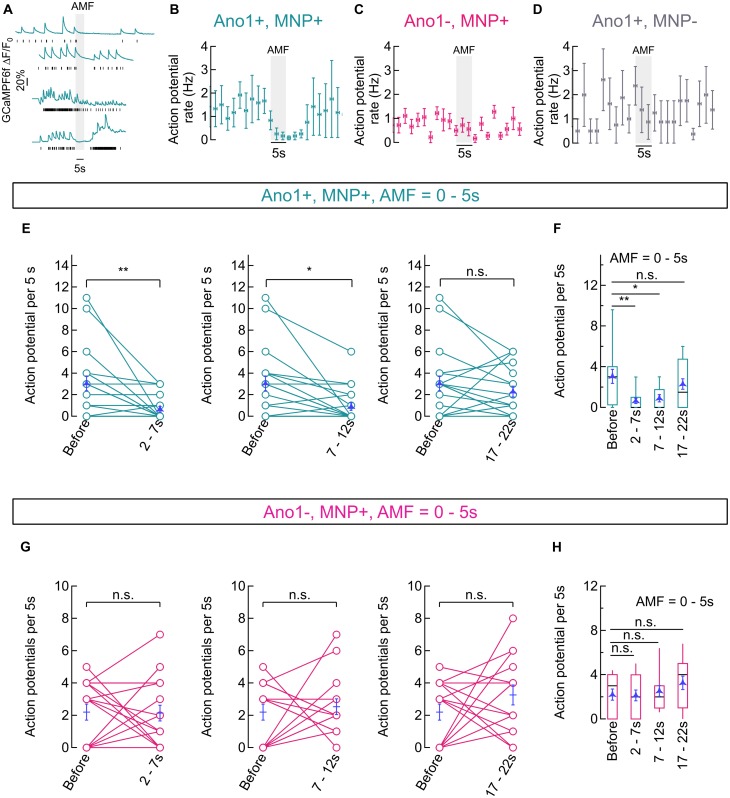
Magneto-thermal silencing of hippocampal neurons. **(A)** Representative traces of GCaMP6f fluorescence intensity averaged over pixels containing the soma of Ano1/TMEM16A^+^, MNP^+^ neurons, exposed to AMF (28.87 ± 1.03 kA/m, 412.5 kHz; gray, solid). Black sticks under each plot indicate respective calculated action potential (AP) events. **(B)** Binned (mean ± SEM, *n* = 9) AP firing rates (binned over 2 s, Hz) of Ano1/TMEM16A^+^, MNP^+^ neurons. Starting temperature was 37°C and AMF (28.87 ± 1.03 kA/m, 412.5 kHz; gray, solid) was applied for 5 s. **(C,D)** Binned (mean ± SEM, *n* = 9) AP firing rates (2 s bins, Hz) of Ano1/TMEM16A^-^, MNP^+^ (*n* = 15, **C**) and Ano1/TMEM16A^+^, MNP^-^ (*n* = 5, **D**) neurons are shown. Starting temperature was 37°C and AMF (28.87 ± 1.03 kA/m, 412.5 kHz; gray, solid) was applied for 5 s in both cases. **(E,F)** Shows the comparison between AP firing rates recorded in 5 s time intervals corresponding to various temperature ranges achieved during magnetothermal membrane heating (and subsequent cooling) in Ano1/TMEM16A^+^, MNP^+^ neurons (see **Figure 5B**). Connected plots in E compares AP firing rates at different time bins after AMF application (starting at 0 s) with AP rates before AMF, in Ano1/TMEM16A^+^, MNP^+^ neurons. Overlaid blue markers show mean ± SEM (*n* = 20). AP rates *before* AMF application (37°C) was 3.05 ± 0.68 AP per 5 s; AP rates during *2–7 s* (38–40°C) was 0.65 ± 0.25 AP per 5 s; AP rates during *7–12 s* (38–39°C) was 0.90 ± 0.35 AP per 5 s; and AP rates during *17–22 s* (37–38°C) was 2.30 ± 0.51 AP per 5 s. Significance level of each comparison is indicated alongside. The firing rate suppression was significant in *2–7 s* and *7–12 s* intervals (*p* = 0.0032 and 0.0094, respectively), while insignificant rate change was observed in the *17–22 s* bin (*p* = 0.3858), unpaired *T*-test. Box and whisker plot in **(F)** summarizes the results in **(E)**. Boxes span from 2nd to 3rd quartile (box top, 75% and box bottom, 25%), while whiskers indicate 10th and 90th percentiles. The black lines dividing the boxes indicate the median, while mean ± SEM values are overlaid in blue. **(G,H)** Shows the comparison between AP firing rates recorded in 5 s time intervals corresponding to various temperature ranges achieved during Magnetothermal membrane heating (and subsequent cooling) in Ano1/TMEM16A^-^, MNP^+^ neurons. Connected plots in **(G)** compares AP firing rates at different time bins after AMF application (starting at 0 s) with AP rates before AMF, in Ano1/TMEM16A^-^, MNP^+^ neurons. Overlaid blue markers show mean ± SEM (*n* = 15). AP rates *before* AMF application (37°C) was 2.20 ± 0.50 AP per 5 s; AP rates during *2–7 s* (38–40°C) was 2.13 ± 0.48 AP per 5 s; AP rates during *7–12 s* (38–39°C) was 2.53 ± 0.49 AP per 5 s; and AP rates during *17–22 s* (37–38°C) was 3.53 ± 0.61 AP per 5 s. Significance level of each comparison is indicated alongside. The firing rate change was significant in all cases: *2–7 s, p* = 0.9245; *7–12 s*, *p* = 0.6364; and *17–22 s*, *p* = 0.1044, unpaired *T*-test. Box and whisker plot in **(H)** summarizes the results in **(G)**. Boxes span from 2nd to 3rd quartile (box top, 75% and box bottom, 25%), while whiskers indicate 10th and 90th percentiles. The black lines dividing the boxes indicate the median, while mean ± SEM values are overlaid in blue. ^∗^*p* < 0.05, ^∗∗^*p* < 0.005.

Alternating magnetic fields (28.87 ± 1.03 kA/m, 412.5 kHz) was applied for 5 s intervals using a magnetic hyperthermia coil placed over the delrin sample holder containing the cells. Autofocus stabilized GCaMP6f fluorescence images were acquired by a camera at a sampling rate of 10 Hz. Average GCaMP6f fluorescence traces, obtained as mean intensity of the pixels spanning the soma of neurons clearly showed suppression of Ca^2+^ influx upon AMF application in Ano1/TMEM16A^+^ neurons (**Figure 6A**). Computed AP events (shown with black bars, under each GCaMP6f fluorescence trace, **Figure 6A**) show complete silencing in most cases. The AP events were pooled from Ano1/TMEM16A^+^, MNP^+^ neurons from different cultures (*n* = 9), where cells with different initial spiking rates (at 37°C) were chosen. Firing rates (mean ± SEM, APs per 5 s), binned over 2 s intervals are shown in **Figure 6B**. Similar graphs for Ano1/TMEM16A^-^, MNP^+^ (*n* = 15) and Ano1/TMEM16A^+^, MNP^-^ (*n* = 5) neurons are shown in **Figures 6C,D**, respectively.

To quantify the silencing, visibly achieved only in Ano1/TMEM16A^+^, MNP^+^ neurons, we compared the computed AP firing rates within time-bins (AMF = 28.87 ± 1.03 kA/m, 412.5 kHz; 0–5 s) corresponding to different temperature ranges (as mentioned in the previous section). Firing rates, recorded in the 2–7 s interval (0.65 ± 0.25 AP per 5 s; temperature = 38–40°C), were significantly different from the base value (3.05 ± 0.68 AP per 5 s; temperature 37°C), with *p* = 0.0032. Firing rates, recorded in the 7–12 s interval (0.90 ± 0.35 AP per 5 s; temperature = 38–39°C), were also significantly different from the base value (3.05 ± 0.68 AP per 5 s; temperature 37°C), with *p* = 0.0094. Firing resumed in the 17–22 s interval (2.30 ± 0.51 AP per 5 s; temperature = 37–38°C), with a *p*-value of 0.3858. All *p*-values were calculated by unpaired *T*-test and *n* = 20 in all cases (**Figures 6E,F**).

In contrast, Ano1/TMEM16A^-^, MNP^+^ neurons showed no significant change in firing rates with AMF heating. Firing rate at 37°C, before AMF application was 2.20 ± 0.50 AP per 5 s. During 2–7 s (38–40°C), the rate was 2.13 ± 0.48 AP per 5 s, giving a *p*-value of 0.9245; while a rate of 2.53 ± 0.49 AP per 5 s was seen during the during 7–12 s (38–39°C) duration, with a *p*-value of 0.6364; and the 17–22 s (37–38°C) period gave a rate of 3.53 ± 0.61 AP per 5 s, the *p*-value being 0.1044. All *p*-values were calculated by unpaired *T*-test and *n* = 15 in all cases (**Figures 6G,H**).

#### Suppression Ratio in Magnetothermal Silencing

**FIGURE 7:**
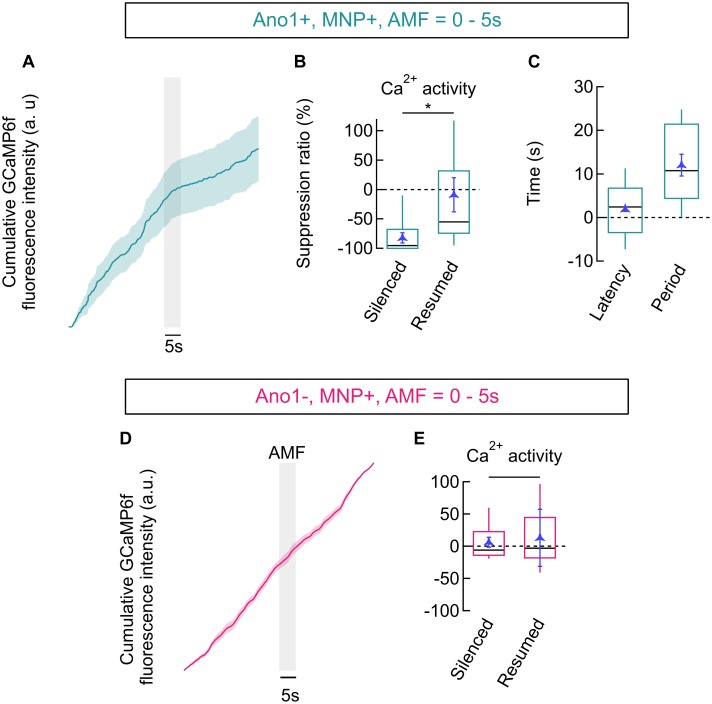
Firing suppression ratio and latency. **(A)** GCaMP6f signal integration (trapezoidal) in Ano1/TMEM16A^+^, MNP^+^ neurons (mean ± SEM, *n* = 12). AMF is indicated by the gray bar. **(B)** Box plot showing ratios of slopes obtained from integration plots in Ano1/TMEM16A^+^, MNP^+^ neurons. Suppression ratio is given as change in slope during suppression (*silenced*) and after the resumption of firing (*resumed*), compared to the initial slope. Ratio obtained during the silenced period was –82.04 ± 8.84% and following resumption was –8.98 ± 28.98% (mean ± SEM). The *p*-value between the *silenced* and *resumed* suppression ratio was 0.028, (*n* = 12, *t*-test). **(C)** Box plot showing the latency of silencing and the period of silencing, following 5 s of AMF in Ano1/TMEM16A^+^, MNP^+^ neurons. Latency was 1.882 ± 0.477 s following the start of AMF application, while the period was 12.05 ± 2.477 s (mean ± SEM). **(D)** GCaMP signal integration (trapezoidal) in Ano1/TMEM16A^-^, MNP^+^ neurons (mean ± SEM, *n* = 16). AMF is indicated by the gray bar. **(E)** Box plot showing ratios of slopes obtained from integration plots in Ano1/TMEM16A^-^, MNP^+^ neurons. The ratios obtained in similar time periods as indicated in **(C)** yield 6.02 ± 7.74% and 12.88 ± 44.20% during the *silenced* and *resumed* periods, respectively. No significant difference between these rates was found (*p* = 0.615, *n* = 16, *t*-test). All AMFs were 28.87 ± 1.03 kA/m r.m.s at 412.5 kHz. Boxes span the second and the third quartile, and the whiskers point at 10th and 90th percentile. Black bars in boxes indicate medians. ^∗^*p* < 0.05.

We quantified the reduction in AP firing in Ano1/TMEM16A^+^, MNP^+^ neurons by calculating the Ca^2+^ activity suppression ratio. To calculate the suppression ratio, we first numerically integrated the bleach corrected, baseline subtracted, normalized GCaMP6f signal. A reduction of the slope of the linear fit of the integrated GCaMP6f signal curve indicates a decline of Ca^2+^ activities, while an increased slope indicates increasing activity (**Figure 2E**). The slopes of the integrated GCaMP6f signal of Ano1/TMEM16A^+^, MNP^+^ neurons were analyzed during three distinct time periods: one prior to AMF application (baseline Ca^2+^ activity rate at 37°C, within -20 to -2 s), one during magnetothermal silencing (lying within 2–22 s, with temperature > 37°C) and a final period, when Ca^2+^ activities resumed to baseline levels (between 22 and 30 s). In **Figure 7A**, a reduced slope is observed in the pooled integration plot, following the AMF application. This corresponds to the silenced period, which is followed by the resumption of Ca^2+^ activities, marked by an increasing slope. During the silenced state, the slope was suppressed by a suppression ratio *S* = 82.04 ± 8.84% compared to the baseline slope. The silenced slope was significantly different from the baseline slope [*p*-value = 0.028 (*n* = 12, unpaired *T*-test)]. The median suppression ratio was 95.45%, while the third quartile was at 67.99%. After normal firing resumed, the slope was indistinguishable from the baseline slope (reduction was -8.98 ± 28.98%) (**Figure 7B**).

Similar analysis on Ano1/TMEM16A^-^, MNP^+^ neurons (**Figure 7D**) showed no significant suppression: suppression ratio of 6.02 ± 12.88% in the period following AMF application (between 2 and 22 s) and 7.74 ± 44.20% long after the AMF was turned off (between 22 and 30 s), (*p*-value = 0.615, *n* = 16, *T*-test) (**Figure 7E**). This ascertains that magnetothermal silencing was unique to Ano1^+^/TMEM16A^+^ neurons.

#### Latency and Duration of Magnetothermal Silencing

To determine latency and duration of magnetothermal silencing of Ano1/TMEM16A^+^, MNP^+^ neurons, we determined the time (with AMF start at time = 0 s) of the intersection points of the sectional fitting lines (indicated by 1 and 2 in **Figure 2E**). Intersection of the slope of baseline activity with the slope of the silenced period defines the beginning of inactivation and hence the *latency of silencing*. Similarly, the intersection of slope during silencing with the slope of the recovered activity defines the end of silencing. We found the latency of silencing in Ano1/TMEM16A^+^, MNP^+^ neurons to be 1.88 ± 0.48 s (after the start of AMF). The end of silencing was observed at 13.93 ± 2.02 s, giving a silencing period of 12.05 ± 2.48 s for 5 s long and 28.87 ± 1.03 kA/m at 412.5 kHz AMF (**Figure 7C**).

## Discussion

In this work, we extended the magnetothermal neuro-modulation technique to silencing neuronal activity, using the thermosensitive chloride channel, Ano1/TMEM16A. Similar to neuronal activation, using TRPV1 ion channels, only 2–3 degrees above the physiological temperature was sufficient to induce significant suppression in firing. For 5 s alternating magnetic field applications, the cells fell silent within 2 s and remained silent for more than 10 s before resuming AP firing. The latency may be tuned by heating efficiency and number of the magnetic nanoparticles attached to the membrane, as well as parameters of the applied alternating magnetic field. The duration can be extended by maintaining an elevated temperature using pulsed AMF application.

Magnetothermal silencing uses the same nanoparticles, targeting method and field application as magnetothermal excitation, just a different ion channel. Hence, the approach can be extended to *in vivo* applications following the procedures of magnetothermal neuronal activation (Munshi et al., 2017). Magnetothermal neuromodulation is minimally invasive, relying only on the delivery of the virus and nanoparticle to the brain region, either done by stereotactic injection or via focused ultrasound-based opening of the blood–brain barrier. Therefore, the preparation work required per animal is small compared to methods requiring implantation of a device. Also, after the wound heals, treated and untreated animals are identical to each other and non-tethered, allowing for unbiased and complex social and behavioral studies.

Generation of the AMF requires integrated high frequency transformer driven resonant capacitor-inductor systems. These are available in form of electronically stabilized, real time programmable commercial units with customizable coils (Ambrell, MSI Automation and others), or as custom builds by research groups (Stauffer et al., 1994; Kallumadil et al., 2012; Subramanian et al., 2016). Applications involving freely moving animals or scaling up to human dimensions, requires large area coils with large currents (Christiansen et al., 2017), but there is no fundamental size limit.

Nanoparticles with higher heating efficiency are still being developed, lowering the AMF requirements (Lee et al., 2011; Chen R. et al., 2013; Zhang et al., 2015; He et al., 2018). An upper limit of AMF power was determined by its effect on biological tissue, which depends on the product of AMF field strength and frequency (Young et al., 1980), while the heating capacity of the magnetic nanoparticles is generally given by the product of the square of field strength and frequency (Rosensweig, 2002). Laboratory synthesized nanoparticles require encapsulation in biopolymers to be biocompatible and to remain stable in suspension inside the body over long periods of time. Already biocompatible alternatives are natural or engineered biosynthesized magnetite nanoparticles from bacteria (Hergt et al., 2005; Fantechi et al., 2014; Elfick et al., 2017; Marcano et al., 2018). Genetically controlled *in situ* syntheses of nanoparticles in neurons could in the future completely remove the need for particle delivery but so far has failed to produce particles showing clear heating (Wesolowski et al., 2009; Pollithy et al., 2011; Stanley et al., 2015).

Thermal activation of Ano1/TMEM16A has been shown to directly induce inward Cl^-^ currents. However, intracellular Ca^2+^ concentration and membrane voltage play a synergistic role in the channels activation, lowering the threshold temperature to near physiological temperatures (Cho et al., 2012). Ano1/TMEM16A has been found in the peripheral nervous system, closely coupled with TRPV1 (transient receptor potential cation channel subfamily V member 1) and is significant in pain enhancement mechanism (Takayama et al., 2015; Oh and Jung, 2016). In the hippocampus, presence of Ano2/TMEM16B, a heat sensitive paralog of Ano1/TMEM16A has been shown to shorten the duration of AP spikes (Huang et al., 2012). Hence, Ano1/TMEM16A overexpression (in the absence of coupled thermosensitive Ca^2+^ conducting channels) can serve as the ideal strategy for inhibitory heat sensitization in central nervous system (CNS) neurons, without eliciting traits of nociception. As Ano1/TMEM16A channels are mammalian ion channels, they target well to the neuronal membrane and are efficient chloride channels (Terashima et al., 2013; Pedemonte and Galietta, 2014). Overexpression of Ano1/TMEM16A in HEK293 cells was found to induce small Cl^-^ currents, resulting from partial activation at 37°C (Schreiber et al., 2018). Applying the channel for silencing in the mammalian brain may benefit from shifting the activation temperature slightly higher, although this was not necessary for TRPV1 which shows also some activation at 37°C when over-expressed. Although the gene encoding Ano1/TMEM16A is too large to be delivered using AAV, we have successfully used Lentivirus to deliver Ano1/TMEM16A to neurons.

## Conclusion

Magnetothermal genetic silencing offers a minimally invasive alternative to optogenetic silencing with the advantages of easy deep tissue penetration of AMF, no requirement for any tether or external marking of the animals, and the possibility for prolonged silencing without the undesired effects of blue light induced photo-toxicity (Grzelak et al., 2001; Dixit and Cyr, 2003; Davies, 2004) and photoinduced tissue heating (Christie et al., 2013), affecting blood flow (Rungta et al., 2017).

## Author Contributions

AP conceived the project. AP, RM, and SQ contributed to design of the research. RM and SQ performed the cell silencing experiments and imaging. RM analyzed the data, wrote the procedures, and prepared the figures. RM and AP wrote the manuscript. All authors reviewed the manuscript.

## Conflict of Interest Statement

The authors declare that the research was conducted in the absence of any commercial or financial relationships that could be construed as a potential conflict of interest.
